# *CpMAX1a*, a Cytochrome P450 Monooxygenase Gene of *Chimonanthus praecox* Regulates Shoot Branching in *Arabidopsis*

**DOI:** 10.3390/ijms231810888

**Published:** 2022-09-17

**Authors:** Haiyuan Zhang, Run Hua, Xia Wang, Huafeng Wu, Hua Ou, Xin Lu, Yan Huang, Daofeng Liu, Shunzhao Sui

**Affiliations:** Chongqing Engineering Research Center for Floriculture, Key Laboratory of Horticulture Science for Southern Mountainous Regions of Ministry of Education, College of Horticulture and Landscape Architecture, Southwest University, Chongqing 400715, China

**Keywords:** strigolactones, *CpMAX1a*, wintersweet, branching

## Abstract

Strigolactones (SLs) are a class of important hormones in the regulation of plant branching. In the model plant *Arabidopsis*, *AtMAX1* encodes a cytochrome P450 protein and is a crucial gene in the strigolactone synthesis pathway. Yet, the regulatory mechanism of *MAX1* in the shoot branching of wintersweet (*Chimonanthus praecox*) remains unclear. Here we identified and isolated three *MAX1* homologous genes, namely *CpMAX1a*, *CpMAX1b*, and *CpMAX1c*. Quantitative real-time PCR (qRT-PCR) revealed the expression of *CpMAX1a* in all tissues, being highest in leaves, whereas *CpMAX1b* was only expressed in stems, while *CpMAX1c* was expressed in both roots and stem tips. However, *CpMAX1a*’s expression decreased significantly after decapitation; hence, we verified its gene function. CpMAX1a was located in *Arabidopsis* chloroplasts. Overexpressing *CpMAX1a* restored the phenotype of the branching mutant *max1–3*, and reduced the rosette branch number, but resulted in no significant phenotypic differences from the wild type. Additionally, expression of *AtBRC1* was significantly upregulated in transgenic lines, indicating that the *CpMAX1a* gene has a function similar to the homologous gene of *Arabidopsis*. In conclusion, our study shows that *CpMAX1a* plays a conserved role in regulating the branch development of wintersweet. This work provides a molecular and theoretical basis for better understanding the branch development of wintersweet.

## 1. Introduction

In plants, the development of side shoots begins in the axillary meristems (AMs) at the leaf axils, with shoot branching usually divided into two developmental stages: the generation of an AM in each leaf axil and the ensuing outward growth process. After their generation, the axillary buds may either remain dormant or grow to form branches [1,2,3]. Branch formation is regulated by a variety of factors, including hormones, developmental status, and the environment, in which phytohormones play an extremely important role [4,5]. Auxins and cytokinins have long been thought to figure prominently in controlling shoot branching, but recent studies suggest that SLs may be involved in plant branching regulation by interacting with auxin and cytokinin as second messengers of auxin [6,7,8,9].

Strigolactones (SLs) were originally isolated from plant root exudates as germination stimulants for root parasitic plants of the Orobanchaceae family, including witchweeds (*Striga* spp.), broomrapes (*Orobanche* and *Phelipanche* spp.), and *Alectra* spp. [10,11]. They were later shown to be indispensable chemical signals for root colonization by arbuscular mycorrhizal fungi (AMF) and therefore considered as beneficial plant metabolites [12]. Furthermore, SLs mediate plant resistance in response to various abiotic stresses (including drought, salinity, temperature) [13,14,15]. Several branching mutants deficient in SLs synthesis and signaling have been well studied: *rms (ramosus) 1–5* mutants in pea *(Pisum sativum)* [16,17,18], *max (more axillary growth) 1–4* mutants in *Arabidopsis* [19,20,21,22], *decreasedapical dominance (dad)* mutants of petunia *(Petunia hybrida)* [23]. Accordingly, SLs or their metabolites are now recognized as a new class of phytohormones whose participation in the inhibition of shoot germination is pivotal.

Presently, the biosynthesis pathway of SLs has been basically elucidated and the key enzymes involved in this pathway identified. Specifically, the carotenoid isomerase DWARF27 (D27) converts all-*trans*-β-carotene to 9-*cis*-β-carotene [24,25], after which CAROTENOID CLEAV AGE DIOXYGENASE 7 (CCD7) catalyzes the cleavage of 9-*cis*-β-carotene to form 9-*cis*-β-apo-10′-carotenal, whose cleavage and oxidation mediated by CAROTENOID CLEAV AGE DIOXYGENASE 8 (CCD8) then forms carlactone [26,27,28]. The cytochrome P450 oxygenase encoded by *MORE AXILLARY GROWTH 1 (MAX1)* synthesizes the precursor of SLs needed to further synthesize them [29,30]. The α/β hydrolase D14 specifically recognizes and hydrolyzes SLs. Subsequently, the receptor SLs D14 protein undergoes a conformational change to form a SCF protein complex with D3 (MAX2) [31,32,33], and the newly SCF formed complex subsequently mediates the ubiquitin-dependent degradation of the transcriptional repressor D53 (SMXL6, SMXL7, SMXL8) and transduces SLs signaling [34,35]. For the biosynthesis or signaling of SLs, these genes are required to properly regulate plant axillary bud growth and shoot branching.

*MAX1* was first discovered in *Arabidopsis* and acts downstream of the *MAX3* and *MAX4* genes, encoding cytochrome P450 monooxygenase (Cyt P450) to convert CL into carlactonoic acid (CLA) [29]. To date, the *MAX1* homologous gene has been studied in many plant species. In rice, deletion of the *MAX1* homolog cytochrome P450 genes *slb1* and *slb2* led to the reduced secretion of SLs. Both rice genes are able to rescue the Arabidopsis *max1–1* highly branched mutant phenotype, and they increase the production of the SL, ent-2′-epi-5-deoxystrigol when overexpressed in Oryza sativa [36]. In tomato, *MAX1* knockout mutants exhibit a strong multi-branched phenotype and reduced SL content, which suggests the biosynthesis of most SLs is accomplished through *MAX1* [37]. Collectively, these studies suggest that *MAX1* plays a key role in regulating plant branch development by affecting SLs’ synthesis.

However, investigations of MAX1 gene’s functioning in ornamental plants are limited, especially in wintersweet (*Chimonanthus praecox*). As a critical agronomic trait, branching can greatly influence the structure and yield of higher plants. The number of branches in the production of wintersweet will directly affect the yield and ornamental quality of this plant’s fresh cut flowers. Therefore, it is of great significance to study the molecular mechanism of *CpMAX1* for regulating the process of branching in wintersweet. Here, we identified and isolated three *MAX1* homologous genes: *CpMAX1a*, *CpMAX1b*, and *CpMAX1c*. The three genes are highly similar, but *CpMAX1a* is expressed more in axillary buds, so we focused on *CpMAX1a* as the research object. To verify the function of *CpMAX1a*, we heterologously expressed *CpMAX1a* in *Arabidopsis*. The overexpression of *CpMAX1a* restored the multi-branched phenotype of the *Arabidopsis* mutant *max1–3*. Further, the number of branches in *CpMAX1a* overexpressed lines decreased but not significantly, suggesting that *CpMAX1a* plays a conserved role in regulating branch germination. Overall, the results of this study enhance our understanding of the role of *CpMAX1a* in the development of lateral branches in wintersweet and provide a basis for exploring the molecular mechanism of branching in this plant and closely related species.

## 2. Results

### 2.1. CpMAX1a Cloning and Phylogenetic Analysis

**Figure 1:**
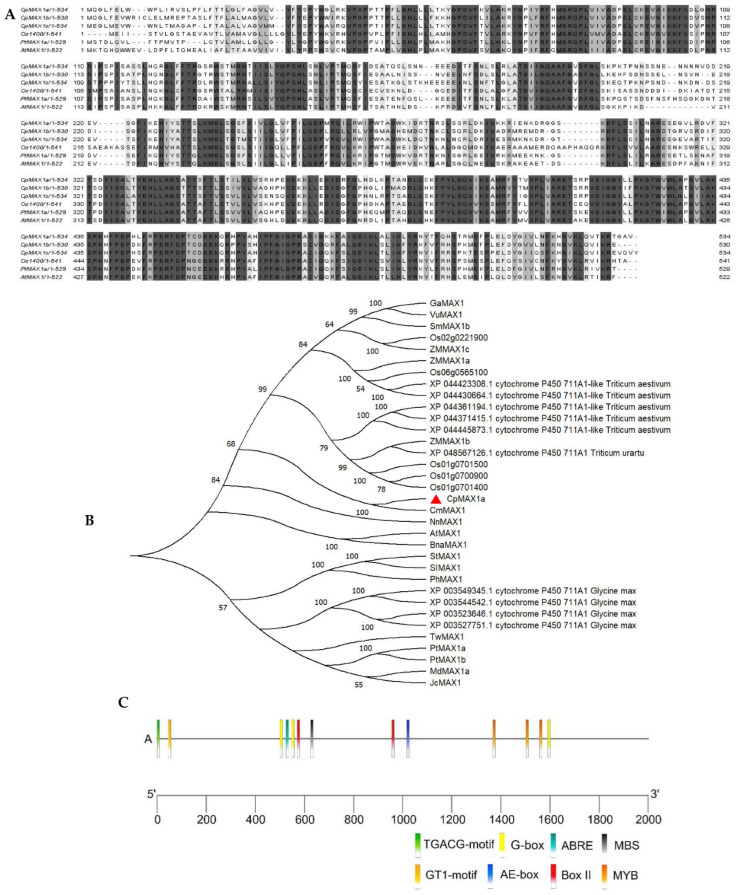
Characterization of *CpMAX-1a* gene in wintersweet. (**A**) Multiple sequence alignment of CpMAX1 proteins with MAX1 proteins from *Oryza sativa* (JX235696), *Populus trichocarpa* (XP_006372016), and *Arabidopsis* (AK316903). Identical amino acids are shaded in black, while similar amino acids are shaded in gray. (**B**) Phylogenetic analysis of *CpMAX-1a* and CYP711As in various plants. MEGA 11 software with the neighbor-joining (NJ) method (1000 bootstrap repeats) was used to reconstruct the phylogenetic tree. (**C**) Cis-acting elements in the promoter of *CpMAX1a*. Accession numbers of the protein sequences and analysis of cis-acting elements of promoters are shown in Tables S2 and S3, respectively.

The *CpMAX1a* was successfully isolated from the leaf samples of wintersweet. The cDNA sequence of *CpMAX1a* was obtained from the wintersweet flower transcriptome database. According to its sequence analysis, *CpMAX1a* has an open reading frame (ORF) of 1605 bp, encoding a protein of 534 amino acids (aa) (Figure 1A), in which leucine (Leu) constitutes the largest proportion (11.8%) of the amino acid composition. Multiple sequence alignment showed that *CpMAX1a* had the highest sequence similarity with the homologous genes *OS1400, AtMAX1, and PtMAX1a* in rice, *Arabidopsis*, and *P. trichocarpa*, respectively (Figure 1B). The phylogenetic tree results indicated that *CpMAX1a* and the *Cinnamomum micranthum* CYP711 subfamily homologous genes clustered together and had the closest genetic relationship. In addition, we cloned the promoter region 2000-bp upstream of *CpMAX1a* gene, using the genomic walking method. This promoter analysis showed that the *CpMAX1a* promoter region harbored seven light-responsive elements (three G-boxes, two Box II, one AE-box, and one GT1-motif), one MeJA responsive element (TGACG-motif), one ABA-responsive element (ABRE), one MYB binding site (MBS), and three stress-responsive elements (MYB), indicating that *CpMAX1a* may be induced by light and stress (Figure 1C).

### 2.2. Expression Pattern of CpMAX1a

**Figure 2:**
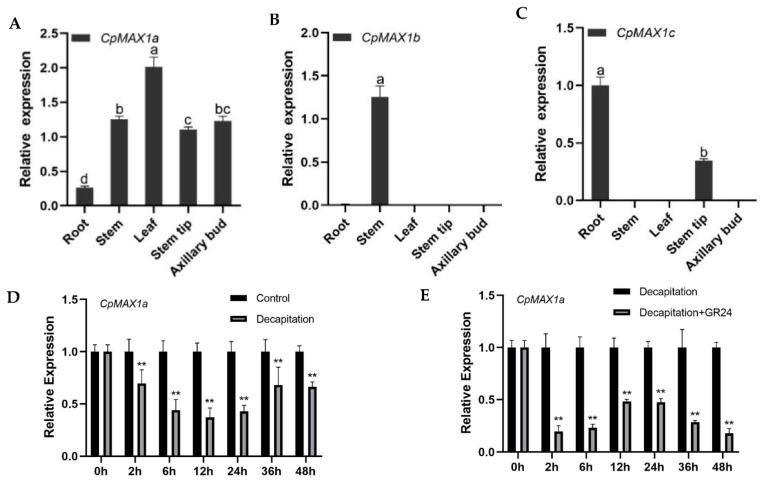
Expression analysis of *CpMAX1* in wintersweet. Expression levels in different tissues of (**A**) *CpMAX1a*, (**B**) *CpMAX1b*, and (**C**) *CpMAX1c* genes. (**D**) Expression levels of *CpMAX1a* in axillary buds after decapitation. (**E**) Expression of *CpMAX1a* in axillary buds after decapitation and the decapitation + GR24 treatment. Data shown are the mean ± standard deviation (SD) of three technical replicates. Different lowercase letters (a–d) above bars indicate significant differences (** *p* < 0.01).

Here we identified and isolated three *MAX1* homologous genes. Among them, *CpMAX1b* is only expressed in stems (Figure 2B), *CpMAX1c* is only expressed in roots and stem tips (Figure 2C), but *CpMAX1a* is expressed in all tissue types (Figure 2A). Based on tissue specificity, it can be concluded that *CpMAX1a* is constitutively regulated, while *CpMAX1b* and *CpMAX1c* are non-constitutively regulated. To analyze the role of *CpMAX1a* in the shoot branching of wintersweet, we used qRT-PCR technology to detect the expression levels of *CpMAX1a* in the six-leaf stage of wintersweet’s axillary buds. Axillary bud samples were collected at 0 h, 2 h, 6 h, 12 h, 24 h, 36 h, and 48 h after decapitation; axillary buds of seedlings without decapitation served as the control. Real-time fluorescence quantitative results showed that the expression of *CpMAX1a* was significantly downregulated in decapitated axillary buds, being lowest at 12 h. Within 48 h after decapitation, *CpMAX1a* was consistently expressed at a low level during the critical period of axillary bud germination (Figure 2D). Further, the GR24 treatment significantly inhibited *CpMAX1a*’s expression. Compared with the decapitation control, the expression level of *CpMAX1a* in axillary buds treated with GR24 remained low throughout the treatment period (Figure 2E).

### 2.3. Subcellular Localization of CpMAX1a

**Figure 3:**
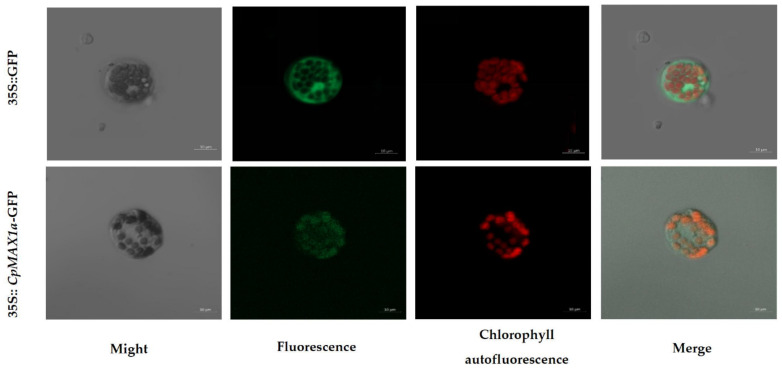
Subcellular localization analysis of GFP-tagged *CpMAX1a*. GFP-tagged *CpMAX1a* genes were expressed in *Arabidopsis* protoplasts. The 35S::GFP construct served as the control. Green coloring indicates the GFP signal; red coloring indicates chlorophyll autofluorescence; yellow indicates the merged signal. Scale bars: 10 µm.

To determine the subcellular localization of the protein encoded by *CpMAX1a*, it was fused to the N-terminus of the GFP (green fluorescent protein) gene. Then 35S::*CpMAX1a*-GFP and 35S::GFP (control) were separately transformed into *Arabidopsis* leaf protoplasts. In the 35S::GFP-transformed protoplasts, the GFP signal was dispersed throughout the cytoplasm, whereas in 35S::*CpMAX1a*-GFP the signal was localized in chloroplasts (Figure 3). Hence, these results suggested that the CpMAX1a protein is localized in chloroplasts.

### 2.4. Effect of Environmental Factors on the Expression of CpMAX1a

**Figure 4:**
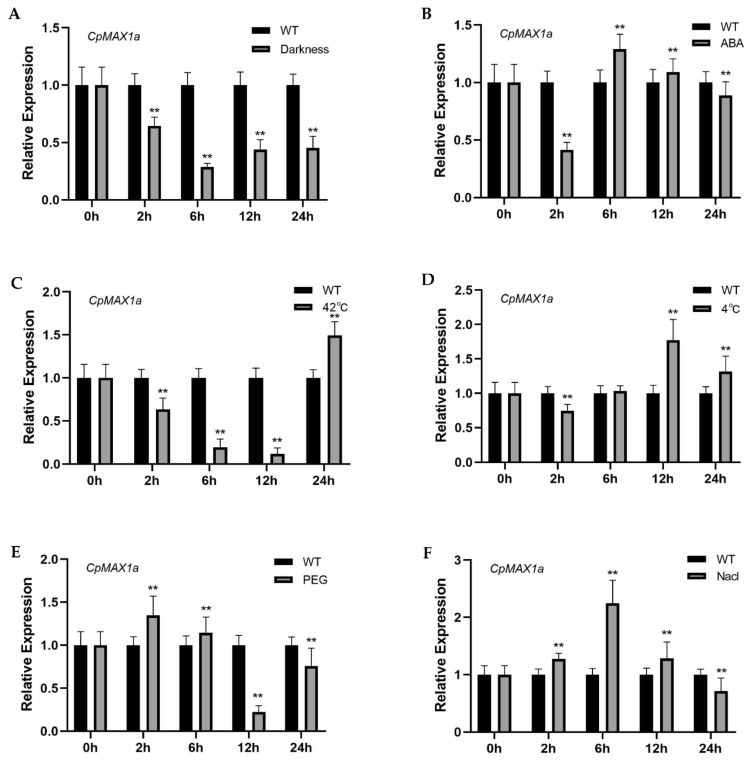
Effect of environmental factors on the expression of *CpMAX1a*. Six-leaf stage wintersweet was exposed to (**A**) Dark treatment, (**B**) 50 µM ABA, (**C**) 42 °C, (**D**) 4 °C, (**E)** 50% PEG-6000, and (**F**) 300 mM NaCl treatments. Data represent the mean of three biological repeats ± SD. Error bars indicate standard deviation. ** *p* < 0.01.

To explore the effect of environmental factors on the expression of *CpMAX1a*, we used qRT-PCR to determine the transcription level of *CpMAX1a* in wintersweet. These treatments were selected based on the cis-acting elements in the *CpMAX1a* promoter region. *CpMAX1a* expression was significantly reduced under dark conditions and was consistently lower than the control (Figure 4A). Under ABA treatment, *CpMAX1a* expression decreased at 2 h, and then increased to the highest value at 12 h (Figure 4B). Under high temperature treatment, *CpMAX1a* expression gradually decreased, but significantly increased at 24 h (Figure 4C). For low temperature treatment, *CpMAX1a* expression decreased at 2 h, and then gradually increased to reach the maximum at 12 h (Figure 4D). In PEG treatment, *CpMAX1a* expression increased significantly at 2 h and then decreased gradually to reach the minimum at 12 h. Interestingly, *CpMAX1a* expression showed an increasing trend again at 24 h (Figure 4E). In Nacl treatment, *CpMAX1a* expression was significantly increased and was significantly higher than the control from 0–12 h (Figure 4F).

### 2.5. Effects of Overexpression of the CpMAX1a Gene on Branching in Arabidopsis

**Figure 5:**
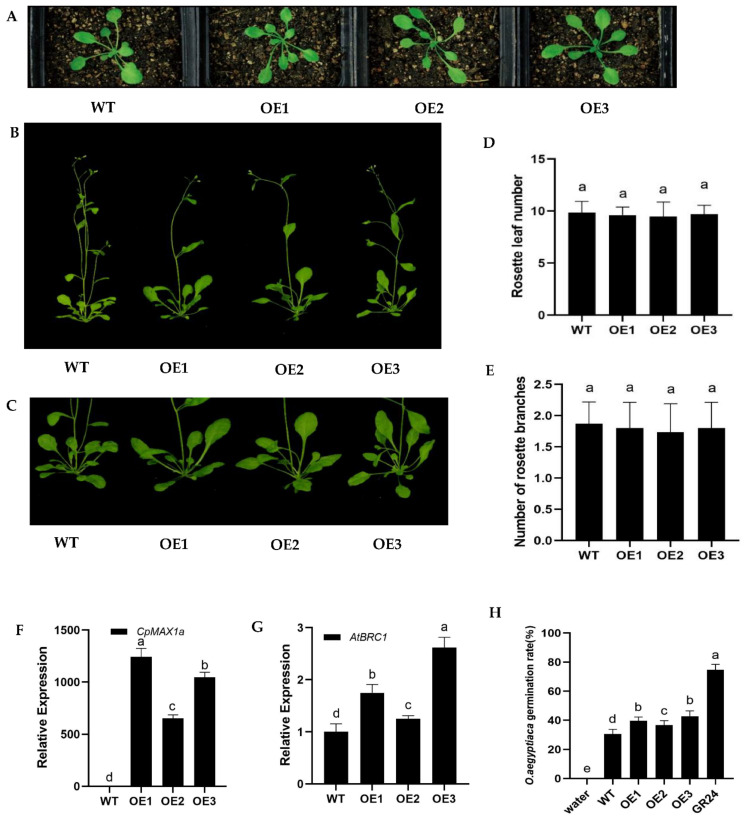
Branching phenotypes of the *CpMAX1a*-OE lines. (**A**) Seedlings grown in soil for 2 weeks. (**B**,**C**) Rosette branching phenotype of *CpMAX1a*-OE plants at 35 days after transplanting. (**D**) Number of rosette leaves in the WT and *CpMAX1a*-OE lines. (**E**) Number of rosette branches in the WT and *CpMAX1a*-OE lines. (**F**) Expression levels of *CpMAX1a* in the transgenic and WT plants. (**G**) Expression levels of *AtBRC1* in the *CpMAX1a*-OE lines and WT. (**H**) The extraction solution of *CpMAX1a*-OE lines induced Orobanche seeds germination experiment; water as a negative control, and GR24 as a positive control. Data shown are the mean of three biological repeats ± standard deviation (SD). Different lowercase letters (a–e) above the bars indicate significant differences (*p* < 0.05).

To investigate the function of the *CpMAX1a* gene, we transformed the 35S:: *CpMAX1a* construct into wild-type (WT) *Arabidopsis*. Six transgenic lines expressing 35S:: *CpMAX1a* were obtained by wetomycin screening and PCR identification. Three pure-hybrid overexpression lines (i.e., OE1, OE2, and OE3) were selected for phenotypic analysis (Figure 5F). We counted the number of rosette leaves of the *CpMAX1a*-OE lines grown under long day (LD) conditions for 15 days and the number of branches at 35 days of growth. These results showed that the mean (±SD) numbers of rosette leaves of the *CpMAX1a-OE* strain were as follows: WT, 9.85 ± 0.72; OE1, 9.62 ± 0.74; OE2, 9.46 ± 1.34; OE3, 9.69 ± 0.82 (Figure 5D); for the number of branches of the *CpMAX1a-OE* strain, the corresponding values were: WT, 1.87 ± 0.34; OE1, 1.8 ± 0.4; OE2, 1.73 ± 0.44; OE3, 1.8 ± 0.4 (Figure 5E). Evidently, the number of rosettes of the *CpMAX1a-OE* strains had slightly fewer rosette leaves and branches than WT strains, but there were no significant differences between them and WT plants. Previous studies have suggested the *MAX1* gene may control *Arabidopsis* meristems by influencing the transcription of *BRC1* [38,39]. Therefore, we examined the expression levels of *AtBRC1* in both WT and *CpMAX1a*-OE strains, finding that *AtBRC1* was more highly expressed in the *CpMAX1a*-OE strains compared to the WT strains. (Figure 5G). The extraction solution of *CpMAX1a*-OE lines induced *Orobanche aegyptiaca* seeds germination experiments showed that the germination rate of *CpMAX1a*-OE lines was higher than that of wt, suggesting that overexpression of *CpMAX1a* in *Arabidopsis* does improve the germination of *Orobanche aegyptiaca* seeds (Figure 5H). These results suggest that *CpMAX1a* may inhibit the growth of *Arabidopsis* rosette branches through the synthesis of SLs by modulating the transcriptional regulation of *AtBRC1*.

### 2.6. Overexpression of the CpMAX1a Gene Restores the Branching Phenotype of Arabidopsis max1 Mutants

**Figure 6:**
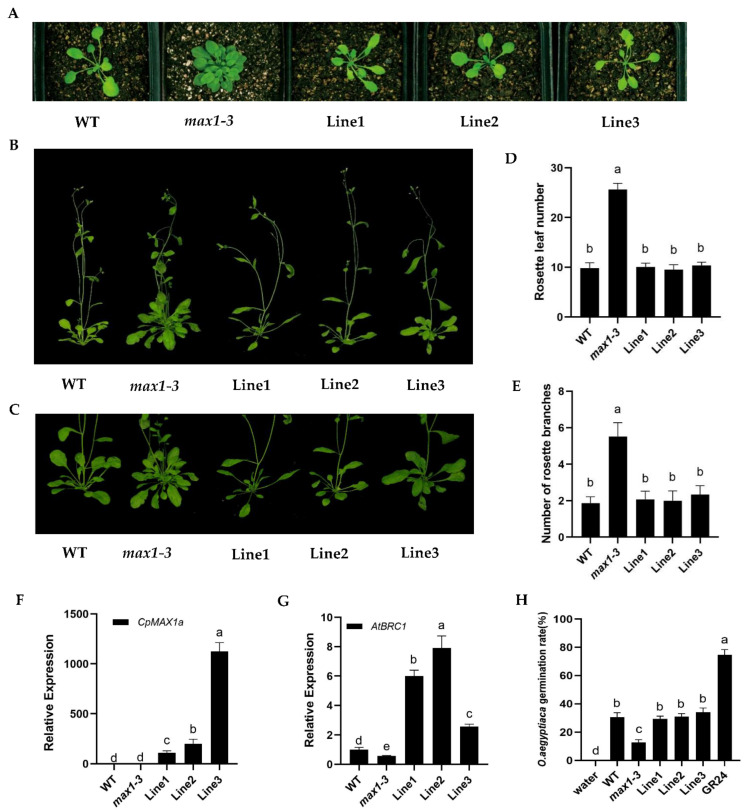
*CpMAX1a* overexpression restores the phenotype of the *Arabidopsis* branching mutant *max1–3*. (**A**) Seedlings grown in soil for 2 weeks. (**B**,**C**) Branching phenotype of the WT, *max1–3* mutant, and restored lines 1–3 grown in soil for 5 weeks. (**D**) Number of rosette leaves and (**E**) number of rosette branches in the WT, *max1–3* mutant, and *CpMAX1a* complementation lines 1–3. (**F**) Expression levels of *CpMAX1a* and (**G**) expression levels of *AtBRC1* in the WT, *max1–3* mutant, and *CpMAX1a* restored lines 1–3. (**H**) The extraction solution of *CpMAX1a* restored lines induced Orobanche seeds germination experiment; water as a negative control, and GR24 as a positive control. Data shown are the mean ± SD of three biological replicates. Different lowercase (a–e) above the bars indicates significant differences (*p* < 0.05).

To further investigate the functioning of the *CpMAX1a* gene, we transformed 35S::*CpMAX1a* into the *Arabidopsis* branch mutant *max1* to obtain nine *CpMAX1a* complementary lines, and selected three independent mutant restorer lines for subsequent experimentation (Figure 6F). We counted the number of rosette leaves of the mutant restorer lines grown under long day (LD) conditions for 15 days and the number of branches at 35 days of growth. Both the number of rosette leaves and branches of the mutant significantly exceeded those of WT, whereas those the restored mutant strain were similar to WT. Mean values for the number of rosette leaves were as follows: WT, 9.85 ± 0.72; *max1–3*, 25.61 ± 1.21; Line1, 10.07 ± 0.73; Line2, 9.54 ± 0.93; Line3, 10.39 ± 0.62 (Figure 6D); for the number of branches, the values were WT, 1.87 ± 0.34; *max1–3*, 5.53 ± 0.71; Line1, 2.07 ± 0.44; Line2, 2 ± 0.52; Line3, 2.33 ± 0.47 (Figure 6E). In addition, we also examined the expression levels of *AtBRC1*, a key gene in the controlling of branching, in the WT lines, mutant lines, and complementary lines. These results showed that *CpMAX1a* significantly upregulated the expression level of *AtBRC1* in the overexpression and complemented lines (Figure 6G). The extraction solution of *CpMAX1a* restored lines induced *Orobanche aegyptiaca* seeds germination experiment showed that the restored lines and wt germination rate were similar, both being significantly higher than *max1–3* (Figure 6H). Altogether, the above results indicated the function of *CpMAX1a* is relatively conserved in the branch development of wintersweet and that its functioning is achieved by regulating the transcription of *BRC1*.

## 3. Discussion

Here, we isolated and identified three *MAX1* homologous genes—*CpMAX1a*, *CpMAX1b*, and *CpMAX1c*—in wintersweet. Given that these three genes are quite similar, yet *CpMAX1a* undergoes high expression in axillary buds, we focused on studying gene as it may be directly involved in controlling branch development. Multiple sequence alignment revealed that the CpMAX1a protein shared a high sequence similarity with the MAX1 protein sequences of other plants. Further, the cytochrome P450 domain motifs were found in the *CpMAX1a* sequence (Figure 1A), which is consistent with the characteristics of MAX1 protein members. Phylogenetic analysis showed that *CpMAX1a* clustered with the *Cinnamomum micranthum* CYP711 protein, indicating that CpMAX1a is most closely related to AtMAX1 (Figure 1B). We found that subcellular localization of the CpMAX1a protein is in the chloroplasts, thus it was speculated *CpMAX1a* could be involved in the synthesis of SLs in plastids. In addition, analysis of the cis-elements of the *CpMAX1a* promoter indicated it harbors multiple elements related to light and stress responses (Figure 1C). *CpMAX1a* expression was significantly reduced under dark conditions (Figure 4A), indicating that light induces *CpMAX1a* expression, which is consistent with the finding that light induces an increase in the content of solanum endolipid in Arabidopsis [40].In addition, ABA, high temperature, low temperature, Nacl and PEG induce an increase in *CpMAX1a* expression(Figure 4B–F),this result is consistent with that adversity can induce the synthesis of SLs in rice, grapevine, submerged plants, and tomato [41,42,43,44].

To the best of our knowledge, there has been no research published on the SL biosynthesis gene *CpMAX1a* in wintersweet. In this study, we examined the expression level of *CpMAX1a* during branch development after decapitation of wintersweet. Compared with the control group, *CpMAX1a* was significantly downregulated at 2 h post-decapitation and maintained a low level of expression during the critical period of axillary bud germination. Further, when GR24 was applied to decapitated wintersweet axillary buds, the expression of *CpMAX1a* was significantly lower than in the control group, a result consistent with the finding that applying GR24 to *Arabidopsis* and sugar beet leads to the diminished expression of genes related to SLs’ synthesis [45,46]. This probably occurred because the negative feedback regulation caused by the substrate GR24 in excess amounts inhibited the expression of *CpMAX1a*, which lends further support to the inference that *CpMAX1a* is involved in the synthesis of SLs. Therefore, we speculate that *CpMAX1a* may inhibit the axillary buds’ growth of wintersweet by regulating the synthesis of SLs. The tissue-specific expression pattern of the *CpMAX1a* gene in wintersweet differs from that of other plants’ homologous genes. In *Arabidopsis*, rice, and tomato, their corresponding *AtMAX1*, *OsMAX1a*, *OsMAX1e*, and *SIMAX1* genes were mainly expressed in the roots and stems [37,47]. In rapeseed (*Brassica napus* L.), the expression of *BnaMAX1* was greatest in axillary buds, followed by the roots [48], though in chrysanthemum its *CmMAX1* gene was highly expressed in leaves, axillary buds, and stems, as well as roots [49]. Here, the *CpMAX1a* gene of wintersweet was expressed most in the leaves and stems, but least expressed in the roots (Figure 2A). Differential expression patterns of *MAX1* in true cotyledons versus monocotyledons suggest that the regulation of branching by SLs is a species-specific process.

To test our speculations and to better understand the functioning of *CpMAX1a*, we heterologously expressed *CpMAX1a* in *Arabidopsis*. We found that in the *CpMAX1a*-OE strains, the number of rosette leaves and rosette branches were not inhibited as expected (Figure 5D,E). Therefore, we performed experiments to induce Orobanche seeds germination by extracts of the *CpMAX1a*-OE strains. The experimental results showed that extracts from the *CpMAX1a*-OE strains indeed promotes the germination, but not to the same extent compared to the exogenous GR24 application(Figure 5H), this means that *CpMAX1a* was involved in the biosynthesis of SLs but did not significantly increase the SLs content, which may also be the reason why overexpression of *CpMAX1a* in *Arabidopsis* did not inhibit branching. When there are not enough SLs in *Arabidopsis* to inhibit auxin transport, rosette branching is not significantly inhibited [9].In addition, overexpression of *CpMAX1a* in *Arabidopsis* could not significantly increase SLs content may be that the complex genetic backgrounds of perennial woody plants and herbaceous plants may also be different, and exogenous expression may not accurately reflect the phenotype of the species studied. Numerous *MAX1* knockout experiments have demonstrated that the functional deletion of the *MAX1* gene results in more branches in *Arabidopsis* [50], tomato [37], rapeseed [48], and rice [47]. Moreover, under the control of the cauliflower mosaic virus (CaMV) 35S promoter, rapeseed *BnaMAX1* was overexpressed in the *Arabidopsis MAX1* branching mutant, restoring the multibranching phenotype [48], and likewise, overexpression of chrysanthemum *CmMAX1* in the *Arabidopsis max1–1* mutant restored its multibranching phenotype [49]. This would suggest *MAX1* plays a conserved role in the control of branch development. To test this conjecture, we performed complementation experiments with the *Arabidopsis max1* mutant, finding here that the *CpMAX1a* gene was indeed capable of restoring the multibranching phenotype of the Arabidopsis mutant *max1–3* (Figure 6B); the extraction solution of *CpMAX1a* restored lines induced *Orobanche aegyptiaca* seeds germination experiments showed that Overexpression of *CpMAX1a* increased the content of SLs in *max1–3* mutants(Figure 6H); hence, it is likely the regulation of plant branching by *CpMAX1a* is conserved in wintersweet. To further analyze the *CpMAX1a* action pathway, we investigated the expression of the gene *BRC1*, which belongs to the TCP gene family and is known for being central how a variety of environmental and developmental factors function locally to inhibit branching [38,51,52]. *BRC1* reportedly acts the downstream of SLs to encode a key transcription factor that inhibits shoot growth, and treatment with SLs can upregulate the expression of *BRC1* [53,54]. The effect of *MAX1* on branching is mainly attributable to the transcriptional control of *BRC1*. Here. we analyzed the relative expression levels of *AtBRC1* in the overexpression and recovery mutant lines, finding them significantly upregulated in both, which strongly suggests that *CpMAX1a* suppresses axillary buds growth in *Arabidopsis* by upregulating its downstream gene *BRC1*. Therefore, we conclude that the role of *CpMAX1a* is conserved in the control of branch development in wintersweet.

As a winter woody ornamental plant, wintersweet’s branching structure confers to it substantial ornamental value. So far, the *CpCCD7* and *CpCCD8* genes have been identified in the SLs’ synthesis pathway in wintersweet [39], but the downstream *CpMAX1a* had not been identified or reported on yet. The uniqueness of wintersweet’s growth and development makes it imperative to understand the contribution of CpMAX1a towards regulating branch development. In this study, we isolated *CpMAX1a* and analyzed its expression pattern and functional characteristics. *CpMAX1a* expression was downregulated during branching in wintersweet, indicating that it negatively regulates axillary buds’ growth in wintersweet. Overexpression of *CpMAX1a*, however, restored the multibranched phenotype of branched mutant *max1–3* and up-regulated the *AtBRC1* gene. These results suggest *CpMAX1a* plays a conserved role in the regulation of wintersweet branching via the regulation of the downstream *BRC1* gene. This study strengthens our understanding of the homologous genes of *CpMAX1a* in wintersweet and provides a timely basis for us to further study the molecular regulatory mechanism of the *CpMAX1a* gene.

## 4. Materials and Methods

### 4.1. Plants

The ‘Suxin’ wintersweet cultivar was used, whose seeds were collected from the campus of Southwest University and sown in the Floriculture Laboratory of Southwest University, China. To analyze the expression pattern of the gene *CpMAX1a* in wintersweet, the roots, stems, leaves, shoot tips, and axillary buds were each collected. These tissues were rapidly frozen in liquid nitrogen once collected. Three biological replicates were obtained for each tissue type sample.

Wild-type *Arabidopsis* (ecotype Columbia−0) and *Arabidopsis max1* (SALK-209654C, code N2105389) purchased from The Arabidopsis Information Resource (TAIR) database were used for the plant transformations. *Arabidopsis* seeds were sown on solid Murashige and Skoog (MS) medium. After vernalization at 4 °C for 3 days, the seedlings were transferred to a constant temperature culture room (22 °C, a 16-h:8-h light:dark photoperiod, 200 Lux light leevl, relative humidity of 70%). When they had four fully expanded leaves, each seedling was transferred to a nutrient bowl (vermiculite:charcoal ratio of 1:1). The results of PCR detection of *CpMAX1a* overexpression in *Arabidopsis* strains are shown in: Figure S2. *Orobanche aegyptiaca* seeds were presented by Prof. Zhao of Shihezi University. *Orobanche aegyptiaca* seed germination experiments were referred to Tsuchiya, Yuichiro et al. [55]. *Arabidopsis* seedlings grown for about 10 days were transplanted into 0.1xMS liquid medium with small glass beads, after 10 days of growth, all contents were washed three times with water, followed by three extractions in batches with ethyl acetate and finally concentrated. After complete evaporation of the solvent, acetone was added according to the fresh weight of *Arabidopsis*, with 10 µL acetone per 1 g. The concentrated root secretion, diluted with water and used for germination of *Orobanche aegyptiaca* seeds. After the *Orobanch* seeds were incubated for 3 days at 25 °C in dark and humid environment, the appropriate volume of root secretion was added and the number of germinations was counted after incubation again at 25 °C in dark environment for 10 days.

### 4.2. Cloning of CpMAX1a Gene

Total RNA was extracted from wintersweet leaves by following the manufacturing instructions of the EASYspin Plant RNA Rapid Extraction Kit (Aidlab, Beijing, China). To ensure their quality and quantity, the RNA extracts were visualized by 1% agarose gel electrophoresis and absorbance measured by a Nanodrop ND-1000 spectrophotometer (Thermo Fisher Scientific, Wilmington, MA, USA) at 260 nm and 280 nm optical densities for quality control (purity) and concentration determination. Synthesis of the cDNA first strand was conducted using the PrimeScript™ RT reagent Kit with the gDNA Eraser (TaKaRa, Japan) reverse transcription kit. The first-step reaction was run in a 10-µL system that contained 2 µL of 5× gDNA Eraser Buffer, 1 µL of gDNA Eraser, and 1 µg of total RNA. Next, the PCR was performed at 42 °C for 5 min. The first strand of cDNA was synthesized by reverse transcriptase in a 20-µL system containing 10 µL of the reaction solution from the first-step reaction, 1 µL of PrimeScript RT Enzyme MixI, 4 µL of RT Primer Mix, 4 µL of 5× PrimeScript Buffer 2, and 1 µL of RNase Free ddH_2_O; then, the PCR was implemented at 37 °C for 15 min, then at 80 °C for 5 s.

Specific primers were designed for *CpMAX1a*-F/R (Table S1) using Primer Premier 5.0 software, and the *CpMAX1a* gene was amplified from leaf cDNA according to the instructions of the TransStart FastPfu kit (TransGen Biotech, Beijing, China). The PCR procedure consisted of an initial preheating step at 95 °C for 5 min, followed by 30 cycles of denaturation at 95 °C for 30 s, annealing at 56 °C for 30 s, and an extension at 72 °C for 1 min 45 sec, with a final extension carried out at 72 °C for 10 min. The ensuing PCR products were separated by 1% agarose gel electrophoresis, and then the target DNA fragments were recovered using an agarose gel recovery kit (Both, Hangzhou, China) according to the manufacturer’s instructions. The amplified products were ligated to the cloning vector pMD19-T (Takara, Dalian, China) and sequenced externally, by the TsingKe Company (TsingKe, Chengdu, China).

The *CpMAX1a* gene promoter sequence was obtained via a chromosome stepping method, this implemented as described in the Universal Genome Walker^TM^ 2.0 User Manual kit. Specific primers (SP1 and SP2; Table S1) were designed according to the ORF (open reading frame) of the *CpMAX1a* gene. For the genomic DNA walking library, the enzyme digestion template was established using these steps: first, digest with the EcoRV blunt-end enzyme, then passivate the digested product, and then ligate with T4-DNA ligase, and connect the upstream and downstream genome walking adapters overnight in a 16 °C-water bath. The PCR reaction in a 25 µL system contained 2.5 µL of 10× buffer, 0.5 µL of dNTPs, 0.5 of µL primer APF1/2, 0.5 µL of primer SP1/2, 0.5 µL of Taq DNA polymerase, 1 µL of DNA, and 19.5 µL of ddH_2_O. The PCR reaction procedure was performed according to the manufacturer’s instructions. The obtained products were separated by 1% agarose gel electrophoresis, the target band was recovered, and the *CpMAX1a* promoter sequence was determined by sequencing.

### 4.3. Bioinformatics Analysis

Multiple sequence alignment analysis was performed using DAMAN 8 software. Amino acid sequences encoded by the *CpMAX1a* gene and its heterospecific homologs were analyzed via alignment, using the NCBI’s (National Center for Biotechnology Information) online BlastP tool: https://blast.ncbi.nlm.nih.gov/Blast.cgi (accessed on 11 October 2021), and subjected to an evolutionary tree analysis in MEGA 11 software, in addition to performing a protein basic information analysis online using ProtParam at http://web.expasy.org/protparam/ (accessed on 25 April 2022). The obtained promoter sequence for the *CpMAX1a* gene of wintersweet was analyzed using a bioinformatics online tool, PlantCARE http://bioinformatics.psb.ugent.be/webtools/plantcare/htmL (accessed on 20 May 2022), to predict the potential cis-regulatory elements and transcription start site of this promoter.

### 4.4. Quantitative Real-Time PCR (qRT-PCR)

The expression pattern of the *CpMAX1a* gene was analyzed in different tissues of wintersweet. Total RNA extraction and cDNA first strand synthesis were performed on wintersweet tissues (as described in Section 2.2). Gene expression was analyzed by qRT-PCR by using the SsoFastTMEvaGreen Supermix and the Bio-Rad CFX96 system. The Ssofast EvaGreen Supermix (50 × 20 µL reactions) includes a 2× reaction buffer containing dNTPs, an Sso7d fusion polymerase, MgCl_2_, EvaGreen dye, and a stabilizer [according to the manufacturer’s instructions]. Each 10 µL reaction mixture contained 5 µL of the Ssofast EvaGreen Supermix (Bio RAD, Hercules, CA, USA), 0.5 µL of each gene specific primer, and 3.5 µL of nuclease-free water. The qRT-PCR was run under the following conditions: 95 °C for 3 min, followed by 40 cycles of 95 °C for 5 s, 60 °C for 5 s and 72 °C for 5 s, and a melt cycle spanning 65 °C to 95 °C. There were three biological replicates and three technical replicates per tissue type sample. The *CpActin* gene served as an internal reference gene for wintersweet [56]. The qRT-PCR primers for *CpMAX1a* (RT-*CpMAX1a*-F/R) can be found in Table S1; all primers were designed in Primer Premier 5.0 software.

### 4.5. Construction of Expression Vectors

The *CpMAX1a* gene was fused into the *pCAMBIA1300*-GFP vector harboring CaMV35S, via a DNA recombination reaction, to generate the 35S::*CpMAX1a*-GFP. Specific primers (Sacl-*CpMAX1a*-F/BamH1-*CpMAX1a*-R) (Table S1) used for this can be found in Table S1, and the plasmid map of the *pCAMBIA1300*-GFP vector is shown in Figure S1.

### 4.6. Generation and Screening of Transgenic Arabidopsis

The 35S::*CpMAX1a*-GFP recombinant plasmid was transformed into wild-type *Arabidopsis* as well as *Arabidopsis max1* plants by inflorescence infestation, to obtain the transgenic strains [57].The generation seeds were sown in MS medium that contained thaumatin (Hyg, 25 mg/L) resistance for their transgenic seed screening. Transgenic positive plants and wild-type plants were transferred into nutrient bowls after that screening, and then sampled at ca. 35 days of incubation. Their leaf DNA was extracted by CTAB, and this extracted DNA used for the PCR amplification of *CpMAX1a*-F/R with specific primers; the PCR procedure included an initial preheating step at 95 °C for 5 min, followed by 27 denaturation cycles run of 95 °C for 30 s, annealing at 56 °C for 30 s, and an extension at 72 °C for 1 min 40 s, followed by a final extension time at 72 °C for 10 min. The ensuing PCR products were detected by 1% gel electrophoresis to confirm *CpMAX1a*’s insertion into the transgenic plants.

The qRT-PCR was used to detect and determine the expression levels of *CpMAX1a* in transgenic strains with *AtActin* serving as the internal reference gene. Three T-3 generation transgenic lines were selected for phenotypic observations and statistics. The numbers of rosette leaf branches and stem branches were counted 35 days after transplanting.

### 4.7. Subcellular Localization of CpMAX1a

To determine the subcellular localization of the protein CpMAX1a, the ORFs of *CpMAX1a* without its stop codon were cloned into the *pCAMBIA1300* vector by using the SacI and BamHI sites. The obtained plasmid 35S:*CpMAX1a*-GFP or an empty vector was then introduced. Protoplasts were transformed using the Arabidopsis Protoplast Preparation and Transformation Kit (Coolaber, Beijing, China), according to the manufacturer’s instructions, and the GFP signal observed by confocal microscopy (Zeiss, Germany). Primers used for the plasmid construction are listed in Table S2.

### 4.8. Statistical Analysis

Data were analyzed by one-way analysis of variance (ANOVA) and Duncan’s test using IBM SPSS 22 software (SPSS, Chicago, IL, USA) and GraphPad Prism 8 (Insightful Science, Republic of Chile), for which *p* < 0.05 and *p* < 0.01 were considered statistically significant and highly significant, respectively.

## Data Availability

Not applicable.

## Supplementary Materials

The following supporting information can be downloaded at: https://www.mdpi.com/article/10.3390/ijms231810888/s1.
