# Supplementary material for: CpMAX1a, a Cytochrome P450 Monooxygenase Gene of Chimonanthus praecox Regulates Shoot Branching in Arabidopsis
Source: Int J Mol Sci. 2022 Sep 17;23(18):10888. doi: 10.3390/ijms231810888 (PMC9503991; doi:10.3390/ijms231810888)

Table S1. List of primers.

Primer name	Primer sequences (5′-3′)
qCPMAX1a（F）：	CCTGATAAGGGTCAGTCTGC
qCPMAX1a（R）：	GAAGGTATGCTTCTGTTACC
MAX1a（F）：	ATGCAAGGATTGTTTGAGTTATGGT
MAX1a（R）：	TCAAACGGCACCTGTTCTCTTTGTG
Sacl-*MAX1a*（F）：	CGAGCTCATGCAAGGATTGTTTGAGTTATGGT
BamH1-*MAX1a*（R）：	CGGGATCC CACAAAGAGAACAGGTGCCGTT
RT-*CpActin*-F	AGGCTAAGATTCAAGACAAGG
RT-*CpActin*-R	TTGGTCGCAGCTGATTGCTGTG
RT-*CpTublin*-F	GTGCATCTCTATCCACATCG
RT-*CpTublin*-R	CAAGCTTCCTTATGCGATCC
AtActin-F	CTTCGTCTTCCACTTCAG
AtActin-R	ATCATACCAGTCTCAACAC
Sp1	GCAAGAAAGGGAAGAATGAATA
Sp2	ATCCGAACCAAATTCCAAGC

Table S2. Analysis of cis-acting elements of promoters.

No.	Name of elements	Amount	Sequence	function
1	ABRE	1	ACGTG	cis-acting element involved in the abscisic acid responsiveness
2	AE-box	1	AGAAACAA	part of a module for light response
3	ARE	2	AAACCA	cis-acting regulatory element essential for the anaerobic induction
4	Box II	2	ACACGTAGA/TGGTAATAA	part of a light responsive element
5	CAT-box	1	GCCACT	cis-acting regulatory element related to meristem expression
6	G-box	3	TACGTG/TAACACGTAG /CACGAC	cis-acting regulatory element involved in light responsiveness
7	GT1-motif	1	GGTTAA	light responsive element
8	MBS	1	CAACTG	MYB binding site involved in drought-inducibility
9	MYB	3	TAACCA/CAACCA	
10	TGACG-motif	1	TGACG	cis-acting regulatory element involved in the MeJA-responsiveness

Table S3. Names and accession numbers used for phylogenetic analysis

Name	Accession number
StMAX1	XP_006351579.1
GaMAX1	BCB01554.1
SmMAX1b	XP_024532645.1
VuMAX1	XP_027918387.1
MdMAX1	XP_ 008393629.2
PtMAX1a	XP_ 006372016.1
TwMAX1	XP_038700752.1
PtMAX1b	XP_ 006382011.1
SIMAX1	XM 004245037
PhMAX1	HM117628.1
AtMAX1	Ak316903
BnaMAX1	XP_013734973.2
ZmMAX1b	LC318663
Os1500	JX566699
Os900	JX235697
Os1400	JX235696
ZmMAX1a	FJ957947
Os5100	JX566698
ZmMAX1c	NM 001152340
Os1900	BAS77710.1
JcMAX1	NW_023591404.1
CmMAX1	RWR72590.1
SmMAX1	XP_024532645.1
cytochrome P450 711A1 [Glycine max]	XP_003549345.1
cytochrome P450 711A1 [Glycine max]	XP_003544542.1
cytochrome P450 711A1 [Glycine max]	XP_003523646.1
cytochrome P450 711A1 [Glycine max]	XP_003527751.1
cytochrome P450 711A1 [Triticum urartu]	XP_048567126.1
cytochrome P450 711A1-like [Triticum aestivum]	XP_044361194.1
cytochrome P450 711A1-like [Triticum aestivum]	XP_044371415.1
cytochrome P450 711A1-like [Triticum aestivum]	XP_044423308.1
cytochrome P450 711A1-like [Triticum aestivum]	XP_044430664.1
cytochrome P450 711A1-like [Triticum aestivum]	XP_044445873.1

Figure S2. PCR Detection of *CpMAX1a* overexpression *Arabidopsis*. OE-1-6: *CpMAX1a* transgenic *Arabidopsis*; L1-10: *CpMAX1a* transgenic *Arabidopsis* mount(*max1*) ;WT: wild-type Arabidopsis. M: DNA Maker DL2000.

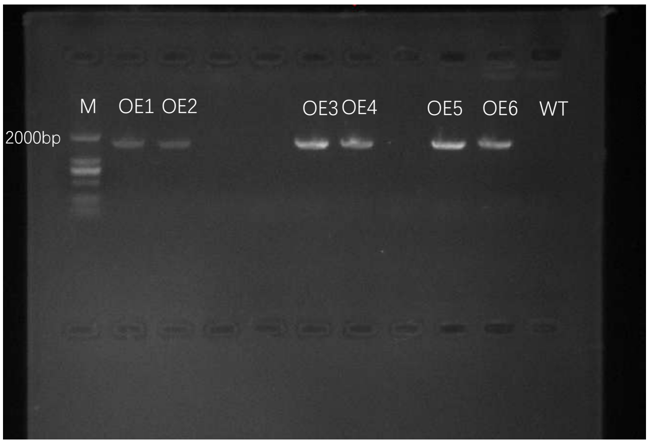

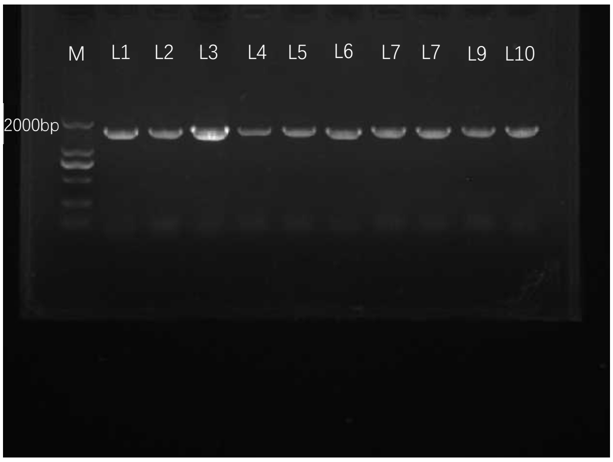

Figure S1. The simple map of the *pCAMBIA1300-GFP* vector.

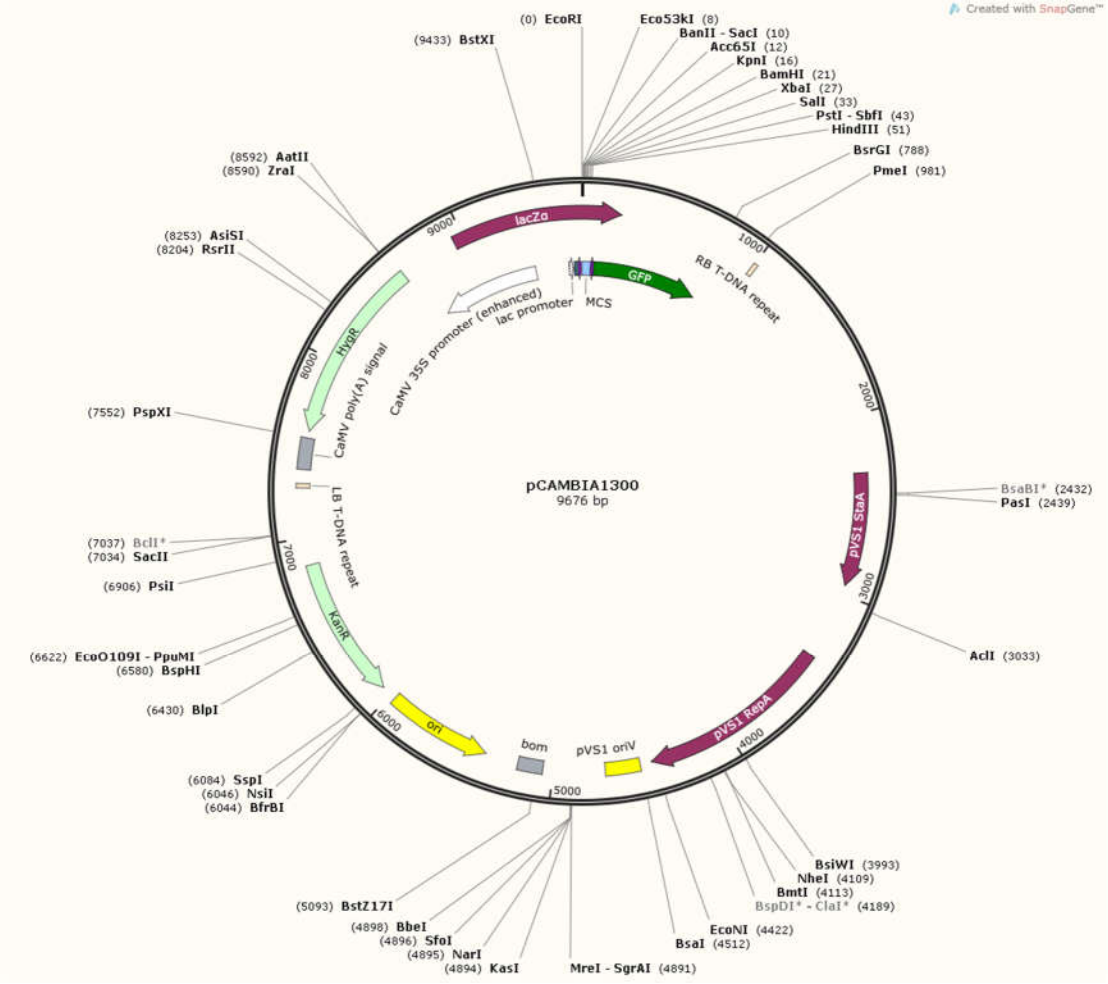

Supplement: Supplementary file 1 [file ijms-23-10888-s001.zip › ijms-1886569-SI.pdf]
